# GLP-1 Receptor Agonist-Associated Weight Loss and Aesthetic Breast Surgery: A Narrative Review and Experience-Based Recommendations for Plastic and Reconstructive Surgeons

**DOI:** 10.1093/asjof/ojag054

**Published:** 2026-03-28

**Authors:** Maurice Y Nahabedian, Anand K Deva, Deeba Ahmed, Paolo Fanzio, Jason Hammer

## Abstract

The use of glucagon-like peptide-1 receptor agonists (GLP-1 RAs) for inducing weight loss has increased in recent years, resulting in a parallel reduction in demand for bariatric surgery. Consequently, the need for patients to undergo aesthetic procedures, including aesthetic breast surgery, following successful GLP-1 RA–induced weight loss is expected to increase while the demand created by postsurgical weight loss is expected to decrease. The objective of this narrative review is to examine the literature on the use of GLP-1 RAs and other mechanisms used for medical weight loss to assist plastic surgeons in achieving optimal aesthetic outcomes with breast surgery after weight loss occurs. To provide an overview of GLP-1 RAs in the context of aesthetic breast surgery, a PubMed literature search was performed using terms such as “glucagon-like peptide-1 receptor agonist,” “GLP-1,” and “breast.” Relevant studies in English published before December 6, 2024 were identified. Experience-based considerations from the authors (M.N. and A.D.) are provided to complement the currently available literature, and 2 case studies of patients who received aesthetic breast surgery following weight loss with GLP-1 RAs are presented. Because drastic changes in breast appearance may occur following weight loss treatment, including ptosis and asymmetry, surgeons should consider overall weight, body composition, and breast aesthetics and counsel patients prior to breast surgery. As our knowledge surrounding the effects of GLP-1 RA use expands, it is likely that surgical protocols and preoperative/postoperative patient counseling recommendations will need to be amended.

**Level of Evidence**: 5 (Therapeutic) 
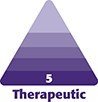

In the United States, there has been growing public interest in glucagon-like peptide-1 receptor agonists (GLP-1 RAs), particularly semaglutide, for inducing weight loss.^1^ In a cross-sectional study of privately insured patients in the United States, the use of metabolic bariatric surgery declined by 25.6%, and the use of GLP-1 RAs as antiobesity medications increased 2-fold from 2022 to 2023.^2^ Although data on prescriptions and administration of GLP-1 RAs as antiobesity treatment are not currently available, reports indicate that the use of these agents for the treatment of type 2 diabetes has increased globally,^3^ including in the United Kingdom,^4^ Australia,^5^ Sweden,^6^ Denmark,^7^ the Netherlands,^8^ and Japan.^9^ Concomitantly, the frequency of bariatric surgery worldwide has declined, with the total number of operations decreasing from 696,191 in 2018 to 604,099 total operations in 2021.^10^

As the demand and usage of GLP-1 RAs for the treatment of patients who are overweight and obese increase, it is important for plastic surgeons to understand the complex relationship between weight loss and body image, in addition to the specific impacts that these treatments may have on the outcome of aesthetic surgery procedures. Following massive weight loss, patients may report increased body image satisfaction as a result of desired physical changes. However, other physical changes, such as excess skin, may lead to body dissatisfaction, and these patients frequently elect to undergo body contouring surgery.^11^ A retrospective analysis of the PearlDiver database found a significant association between use of GLP-1 RAs, including semaglutide and liraglutide, and an increase in aesthetic body contouring surgeries, especially among middle-aged women in the United States.^12^ As GLP-1 RAs are increasingly used, there is expected to be a corresponding increase in the number of patients who have experienced weight loss with these medications seeking aesthetic procedures, including aesthetic breast surgery.

The objective of this narrative review is to examine the literature on the use of GLP-1 RAs and other mechanisms used for medical weight loss to assist plastic surgeons in achieving optimal aesthetic outcomes with breast surgery after weight loss occurs. Experience-based considerations, recommendations, and 2 case studies from 2 surgeons with expertise in the field (M.N., A.D.) are provided to complement the current literature.

## SEARCH STRATEGY

For this narrative review, a literature search of the PubMed database spanning up to December 6, 2024 was conducted to provide an overview of the role of GLP-1 RAs in breast surgery. The following search terms were used: “glucagon-like peptide-1,” “GLP-1,” “glucagon like peptide 1,” “GLP 1,” “dulaglutide,” “liraglutide,” “semaglutide,” “exenatide,” “tirzepatide,” “antiobesity,” “breast,” “bariatric* surg*,” “gastr* band*,” “gastr* bypass,” “gastr* sleeve,” and “aesthetic.” Published articles in English involving preclinical studies, clinical studies (phase 1−4), systematic reviews, meta-analyses, and case reports/case series were selected from the results of the literature search. Abstracts of records resulting from the literature search were screened for relevance, followed by assessment of the full-text articles.

## BRIEF OVERVIEW OF GLP-1 RAS

Glucagon-like peptide-1 receptor agonists have been extensively evaluated in clinical trials, and their mechanism of action is well established.^13^ Glucagon-like peptide-1 receptor agonists decrease glucagon secretion and increase insulin secretion in a glucose-dependent manner, increase satiety, and delay gastric emptying.^13^ These medications lower hemoglobin A1C (HbA1C) levels with a low risk for hypoglycemia, making them an effective treatment option for patients with type 2 diabetes mellitus.^13-16^ Treatment with GLP-1 RAs also results in weight loss, with potential cardiovascular benefits.^13-16^ Based on the published literature,^13-16^ the most common adverse effects following treatment with GLP-1 RAs are injection site reactions and those related to the gastrointestinal system, including nausea, vomiting, and diarrhea. Glucagon-like peptide-1 receptor agonists that are approved by the US Food and Drug Administration (FDA), European Medicine Agency (EMA), and the Therapeutic Goods Administration (TGA) are detailed in Table 1.

**Table 1.** FDA-, EMA-, and TGA-approved GLP-1 Receptor Agonists

GLP-1 receptor agonist	Year of FDA approval	Year of EMA approval	Year of TGA approval	Indication(s)	Dose and frequency	Route of administration	Half-life
Dulaglutide (Trulicity)^17-19^	2014	2014	2015	Adjunct to diet and exercise to improve glycemic control in adults with type 2 diabetes mellitus To reduce the risk of major adverse cardiovascular events in adults with type 2 diabetes mellitus who have established cardiovascular disease or multiple cardiovascular risk factors	0.75−4.5 mg Once weekly	Subcutaneous	5.0 days
Exenatide (Byetta)^20-22^	2005	2006	2007	Adjunct to diet and exercise to improve glycemic control in adults with type 2 diabetes mellitus	5.0−10.0 µg Twice daily	Subcutaneous	2.4 hours
Liraglutide (Victoza)^23-25^	2010	2009	2010	Adjunct to diet and exercise to improve glycemic control in adults with type 2 diabetes mellitus To reduce the risk of major adverse cardiovascular events in adults with type 2 diabetes mellitus and established cardiovascular disease	0.6−1.8 mg Once daily	Subcutaneous	13.0 hours
Lixisenatide (Adlyxin)^26^ (Lyxumia)^27,28^	2016	2013	2013	Adjunct to diet and exercise to improve glycemic control in adults with type 2 diabetes mellitus	10−20 µg Once daily	Subcutaneous	3.0 hours
Semaglutide (Ozempic and Wegovy)^29-32^	2017 (Ozempic) 2021 (Wegovy)	2018 (Ozempic) 2022 (Wegovy)	2019 (Ozempic) 2022 (Wegovy)	Ozempic: Adjunct to diet and exercise to improve glycemic control in adults with type 2 diabetes mellitus Wegovy: To reduce the risk of major adverse cardiovascular events in adults with established cardiovascular disease and either obesity or overweight To reduce excess body weight and maintain weight reduction long term in adults and pediatric patients aged 12 years and older with obesity To treat noncirrhotic metabolic dysfunction-associated steatohepatitis (MASH) with moderate to advanced liver fibrosis	Ozempic: 0.25−1.0 mg Once weekly Wegovy: 0.25−2.4 mg Once weekly	Ozempic: Subcutaneous Wegovy: Subcutaneous	Ozempic: 7.0 days Wegovy: 7.0 days
Tirzepatide^a^ (Mounjaro)^33-35^ (Zepbound)^36^	2022	2022	2022	To reduce excess body weight and maintain weight reduction long term in adults with obesity or adults with overweight in the presence of at least 1 weight-related comorbid condition To treat moderate to severe obstructive sleep apnea in adults with obesity	2.5−15 mg Once weekly	Subcutaneous	5.0−6.0 days

EMA, European Medicines Agency; FDA, Food and Drug Administration; GLP-1, glucagon-like peptide-1; TGA, Therapeutic Goods Administration.

^a^Tirzepatide is a GIP receptor agonist and GLP-1 receptor agonist.

As the use of GLP-1 RAs increases, healthcare appears to be moving toward a consumer-driven environment, especially with respect to these agents.^37^ A cross-sectional study reported that nearly half of the programs sponsoring GLP-1 as a weight loss medication were telemedicine based, and that the majority of these programs were accessed remotely with no in-person interactions.^37^ These trends may come with some risk, as consumers seek to bypass the traditional physician/pharmacy pathway to accessing treatment.^37^ Another cross-sectional study recently reported that websites selling GLP-1 RAs may misinform potential consumers.^38^ Additionally, a qualitative study reported that approximately 42% of websites advertising semaglutide without a prescription belonged to illegal pharmacy operations.^39^ This is an important consideration for surgeons, as their patients may not have consulted with their primary care physician prior to starting a GLP-1 RA for weight loss.

## MASSIVE WEIGHT LOSS AND BREAST AESTHETICS

Breast tissue is primarily composed of fat,^40^ and the reduced volume of fat following massive weight loss, regardless of the method used to lose weight, leads to dramatic changes in the breasts.^41^ Following induced weight loss, including bariatric surgery, the literature reports that breasts may develop severe ptosis, asymmetry, and reduced lateral curvature, as well as loss of upper pole volume.^42,43^ Although not reported in the GLP-1 RA literature, the authors (M.N., A.D.) occasionally encounter patients seeking aesthetic breast surgery following weight loss with GLP-1 RAs that present with wrinkly chest and breast skin, and nipple ptosis. The term “Ozempic breasts,” although not a medical or clinical term, may be used colloquially in reference to changes in breast size or shape due to GLP-1 RA–induced massive weight loss. “Ozempic face” and “Ozempic butt” are similar, informal terms used to describe volume loss and reduced skin elasticity in these body areas that patients may experience following GLP-1 RA–induced massive weight loss.^44,45^ The use of these terms in the mainstream vernacular highlights awareness among the general public that massive weight loss is often accompanied by characteristic changes in appearance.

Reshaping, repositioning, and volumizing the breast following postsurgical weight loss is technically difficult,^43,46^ but also challenging, in part, due to high expectations of the patient.^46^ In a survey of 252 patients who had undergone gastric bypass surgery, 75% of female and 68% of male patients reported that they desired body contouring surgery; the abdomen was the area most reported by patients as requiring body contouring surgery, followed by the breasts and thighs.^47^ Because aesthetic shortcomings can negatively affect a patient's body image and quality of life, it is of the utmost importance that surgeons discuss realistic expectations for patients seeking body contouring surgery during preoperative counseling.^47^

## ASSESSMENT OF WEIGHT STABILITY

There is no consensus on the maintenance of a stable weight prior to aesthetic surgery; however, a clinical study^48^ and published guidelines^49,50^ recommend a minimum of 3-6 months. Although evidence to guide this decision with respect to weight loss with GLP-1 RAs and aesthetic breast surgery is lacking, based on the authors' (M.N., A.D.) clinical experience, we recommend a patient maintain their target weight for a minimum of 3 months prior to aesthetic surgery. Confirmation of this can be sought through collaborative consultations with other health professionals sharing the care of the patient (ie, endocrinologist, dietician, and/or bariatric surgeon). Alternatively, the authors (M.N., A.D.) suggest that a 3- to 6-month gap between initial consultation and surgery may be prudent to ensure that patients are adequately motivated and incentivized prior to undergoing aesthetic surgery to adhere to their program of weight maintenance.

Additional weight loss following aesthetic surgery (>5 kg [>11 lb]) can significantly affect breast surgery, leading to pseudoptosis, loss of volume, and secondary ptosis,^51^ and in the authors' (M.N., A.D.) experiences, can significantly impact implant positioning. More often than not, additional weight loss following aesthetic surgery necessitates further surgical procedures, with the inherent increased risk and cost of this revision surgery to the patient.

Failure of weight loss therapy with resultant weight gain following bariatric surgery^52^ or following use of GLP-1 RAs^53^ can impact aesthetic outcomes of body contouring surgery. A retrospective study of 70 patients who underwent body contouring surgery after massive weight loss reported that those who lost weight with bariatric surgery maintained their weight loss significantly better than those who lost weight by dieting,^54^ which could indicate that the presurgical counseling that patients preparing for bariatric surgery receive is effective for future maintenance of weight loss. Furthermore, several retrospective studies have reported that body contouring surgery following bariatric surgery is associated with significantly better weight loss maintenance compared with those who underwent bariatric surgery without subsequent body contouring surgery.^55-57^ This could be due to patients having improved body image as a result of their aesthetic surgery, increasing their motivation to maintain their weight loss. Weight regain following bariatric surgery is dependent on several factors.^52^ Reasons for weight regain after bariatric surgery include difficulty with dietary adherence (eg, high calorie intake), lack of support, behavioral or psychological issues (eg, diet-related behaviors, such as binge eating, or psychological factors, such as anxiety or depression), and lack of physical activity.^58^ As revisional bariatric surgery potentially increases surgical risk and adverse outcomes,^58^ patients may seek to use GLP-1 RAs if they experience weight regain or inadequate weight loss after bariatric surgery.^59^ A systematic literature review and meta-analysis identified 13 observational studies of 1281 patients who received a GLP-1 RA (liraglutide or semaglutide) from 1 to >5 years post–bariatric surgery; after 1 year of GLP-1 RA use, these patients lost an additional 6% to 8% of their body weight on average.^59^

As with bariatric surgery, there is potential for weight regain following discontinuation of GLP-1 RAs. In a randomized, controlled trial, overweight or obese adults who were treated with semaglutide for 68 weeks experienced a substantial reduction in body weight compared with participants who received placebo treatment; however, subsequent withdrawal of semaglutide led to most of the weight being regained within 1 year.^53^ In another study that investigated whether concomitant exercise and treatment with a GLP-1 RA improved healthy weight maintenance once the GLP-1 RA was discontinued, it was demonstrated that supervised exercise combined with GLP-1 RA treatment prevented weight regain 1 year after treatment discontinuation compared with patients who received GLP-1 RA treatment without supervised exercise.^60^ This finding suggests that exercise after cessation of GLP-1 RA use may be an optimal strategy for maintaining weight loss, although further studies are needed to confirm these results.

## POST–WEIGHT LOSS BODY COMPOSITION CONSIDERATIONS

The effects of GLP-1 RAs on clinically relevant muscle composition and mass changes are still unclear. Similar to weight loss induced by diet or bariatric surgery, weight loss associated with GLP-1 RA treatment is predominantly due to a reduction in fat mass, with some loss in lean body mass.^61^ The change in muscle volume that occurs with GLP-1 RA treatment appears to be commensurate with what is expected based on aging, type 2 diabetes status, and the amount of weight loss^62^; however, more evidence is needed to make a definitive statement.

To ensure perioperative optimization and outcomes, the authors recommend that the surgeon prioritize counseling for patients presenting for aesthetic breast surgery following massive weight loss with a GLP-1 RA; specifically, the patient should be counseled on the need for adequate weight-based protein intake for 2-4 weeks preoperatively and another 4 weeks postoperatively.^45^ In a retrospective review of patients post−bariatric surgery who were undergoing abdominoplasty, adequate protein supplementation decreased the frequency of complications of wound healing compared with patients who did not receive protein supplementation.^63^ In the author's (A.D.) experience, patients who have received GLP-1 RAs for weight loss are treated jointly with endocrinology and/or dietician teams who aid in dietary management. There are several treatments in preclinical or clinical development for a wide variety of patient populations (those with GLP-1 RA–induced weight loss, post-bariatric surgery, muscle wasting disease, and loss of muscle mass with obesity and immobilization) that are aimed at maintaining or improving muscle mass, including recombinant growth hormone,^64^ and an array of targets for muscle health such as activin type II receptor,^65-67^ urocortin (Ucn)2,^68^ and Ucn3.^69^ Further investigation on the effects of GLP-1 RAs on muscle composition, and whether a combination therapy aimed at improving muscle mass and/or adequate protein intake has positive effects on postoperative outcomes, for these patients is needed.

## SURGICAL CONSIDERATIONS FOR AESTHETIC BREAST SURGERY IN PATIENTS FOLLOWING WEIGHT LOSS

Although there have been no reports to date indicating that patients who have experienced massive weight loss from GLP-1 RAs should be treated differently with respect to surgery than other populations who have undergone massive weight loss, including patients who have lost weight via bariatric surgery,^45^ the authors (M.D., A.D.) have compiled their preoperative, intraoperative, and postoperative recommendations for patients seeking aesthetic breast surgery following GLP-1 RA–induced weight loss based on clinical experiences with this patient population (Table 2).

**Table 2.** Preoperative, Intraoperative, and Postoperative Considerations Based on Author Recommendations for Aesthetic Breast Surgery in Patients Who Have Undergone Massive Weight Loss With GLP-1 RAs

Preoperative considerations
Discuss realistic expectations for aesthetic outcomes with patients during preoperative counseling
Ensure at least 3 months of weight stability prior to surgery^48-50^
For patients currently using GLP-1 RAs, they should stop taking them 2-4 weeks prior to surgery to reduce risk of aspiration
Select the breast surgery most appropriate for your patient based on breast volume, shape, and degree of ptosis Discuss risks and possible side effects of surgery during preoperative counseling, as patients with a history of massive weight loss may experience more postoperative complications^70^
Quality of skin (eg, elasticity, stretch marks)
Intraoperative considerations
Anesthetic risk of aspiration due to delayed gastric emptying
Antibiotic prophylaxis
Quality of skin (eg, elasticity, stretch marks)
On table placement of compression garments
Postoperative considerations
DVT prophylaxis for 1 week postoperation (for procedures >3 hours)
Wait at least 1 week to resume GLP-1 RA postoperative use
Fluid management
Dietary management
Wound/scar management (eg, massage, laser, silicone tape)
Compression garments
Return to activity and exercise^a^

DVT, deep vein thrombosis; GLP-1 RA, glucagon-like peptide-1 receptor agonist.

^a^Following aesthetic breast surgery, the patient should walk as soon as they feel able; the patient can resume lower-body exercise 3 weeks postsurgery, light upper-body exercise 4 weeks postsurgery, and normal activity 6 weeks postsurgery.

Of note, case reports have identified a potential link between GLP-1 RAs and aspiration while under general anesthesia,^71,72^ as a result of delayed gastric emptying in patients taking GLP-1 RAs, an important consideration of which anesthesiologists should be aware.^73^ A recent consensus clinical practice recommendation that was endorsed by the Australian Diabetes Society, National Association of Clinical Obesity Services, Gastroenterological Society of Australia, and Australian and New Zealand College of Anesthetists proposed a 24-hour clear fluid diet, followed by standard 6-hour fasting prior to general anesthesia for patients receiving GLP-1 RAs.^74^ The authors (M.N., A.D.) recommend patients discontinue GLP-1 RAs 2-4 weeks prior to surgery to reduce the risk of aspiration.

**Figure 1.**
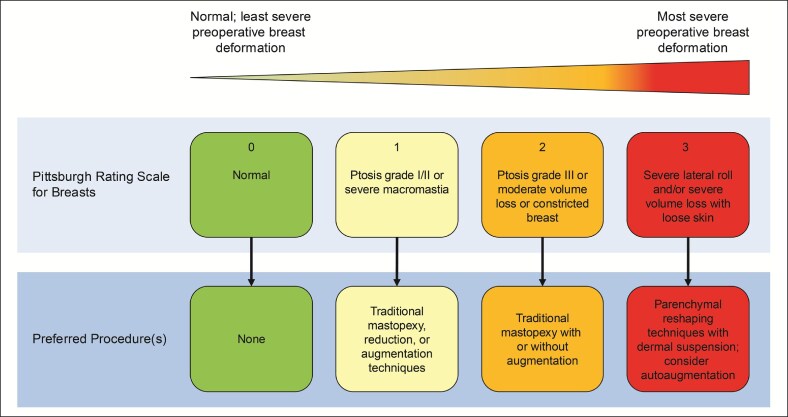
Pittsburgh rating scale and preferred procedures for breasts.^75^ Adapted with permission from Song AY, et al *Semin Plast Surg.* 2006;20:24-29. Copyright 2006 by Thieme Medical Publishers, Inc.

There are a number of breast surgical procedures available to address changes to breast position, volume, and shape resulting from massive weight loss, including mastopexy, breast augmentation, and breast reduction.^46^ Careful assessment of breast volume, shape, degree of ptosis, and asymmetry should form the basis of any surgical planning. The Pittsburgh Rating Scale is a validated instrument that may be helpful for surgeons when preoperatively determining the grade/severity of breast deformity and may aid in selecting which breast procedure is appropriate for a patient following massive weight loss (Figure 1).^75,76^

Ptosis as a result of stretched and inelastic skin and the lowering of the nipple/areolar complex are best corrected by a mastopexy procedure.^42,49^ Following massive weight loss, breast reshaping includes excess skin removal, nipple–areolar complex lifting/lateralization, restoration of breast volume/symmetry, and restoring the fullness of the upper pole and breast medialization.^43^ Some patients may also elect to undergo breast augmentation with or without an implant, or with autologous fat.^49^ A systematic literature review reported that lateral chest wall flaps were the most commonly used autologous tissue for breast reshaping in patients with a history of medical weight loss.^77^ Comparative, randomized studies evaluating the use of autologous flaps combined with mastopexy vs mastopexy combined with the use of implants are needed.^77^ Although surgeons may elect to perform a single-stage augmentation-mastopexy, mastopexy may be performed alone, followed by a second augmentation surgery if desired by the patient.^78^ If an implant is required for volume restoration, published guidelines are available for careful selection of the size, position, and shape of the implant.^79^ The authors (A.D., M.N.) stress the importance of assessing the quality and strength of the soft tissues, breast parenchyma, and skin to ensure that the implant selected can be adequately supported both inferiorly and laterally. In cases where there is inadequate support, the authors recommend a 2-stage procedure, concurrent mesh or dermal matrix, and/or fat grafting without the use of an implant.

A prospective cohort study involving patients who underwent aesthetic breast surgery reported that the incidence of major complications following surgery is typically low, at <2%.^80^ However, performing abdominoplasty and any breast procedure together increased the risk of major complications.^80^ In patients who have experienced massive weight loss, a mastopexy procedure carries some risks, including relapse of ptosis, reduced upper pole volume, and upward migration of the nipple–areolar complex.^42^ These risks should be discussed during preoperative counseling of patients who are seeking aesthetic breast surgery following weight loss, regardless of the weight loss mechanism used (eg, bariatric surgery vs diet and exercise vs GLP-1 RAs). A single-center, retrospective study in patients who underwent autologous breast reconstruction found that patients with a history of bariatric surgery or massive weight loss (>50 lb) experienced significantly more postoperative complications compared with patients without a history of massive weight loss.^70^

Based on published guidelines^49,81^ and clinical studies,^82,83^ plastic surgeons should consider additional risk factors for patients who present for aesthetic breast surgery following weight loss. Patients with high body mass index (BMI; ≥35 kg/m^2^) or poor overall medical condition or who smoke, are being treated with anticoagulants, or have unrealistic expectations for aesthetic outcomes may be poor candidates for aesthetic surgery following massive weight loss.^49^ Patients with high BMIs may experience poorer reconstructive and wound healing outcomes following plastic surgery, including breast reduction or breast reconstruction.^81^ Additionally, clinical studies have reported that obesity is a known risk factor for lymphedema due to its influence on lymph fluid levels and limb volume.^82,83^ A single-center retrospective study in patients undergoing axillary lymph node dissection for breast cancer reported an 86% reduction in the risk of lymphedema in patients who received GLP-1 RAs compared with those who did not receive GLP-1 RAs.^82^ Further research is needed to elucidate the GLP-1 RA mechanism of action in modulating lymphatic function and the potential for reducing the incidence and/or severity of lymphedema.

Seroma is a common complication in body contouring procedures such as abdominoplasty.^84,85^ A retrospective study of 191 patients undergoing abdominoplasty reported that seroma was the most common complication (20.9%); bariatric surgery did not independently impact the risk of complications in these patients.^84^ In the authors' (M.N., A.D.) experiences, patients who have lost weight while taking GLP-1 RAs may have improved healing and fewer incidences of seroma following breast surgery compared with patients who lost weight as a result of bariatric surgery; however, this is an area for future study.

## CASE PRESENTATIONS

### Case Study 1

**Figure 2.**
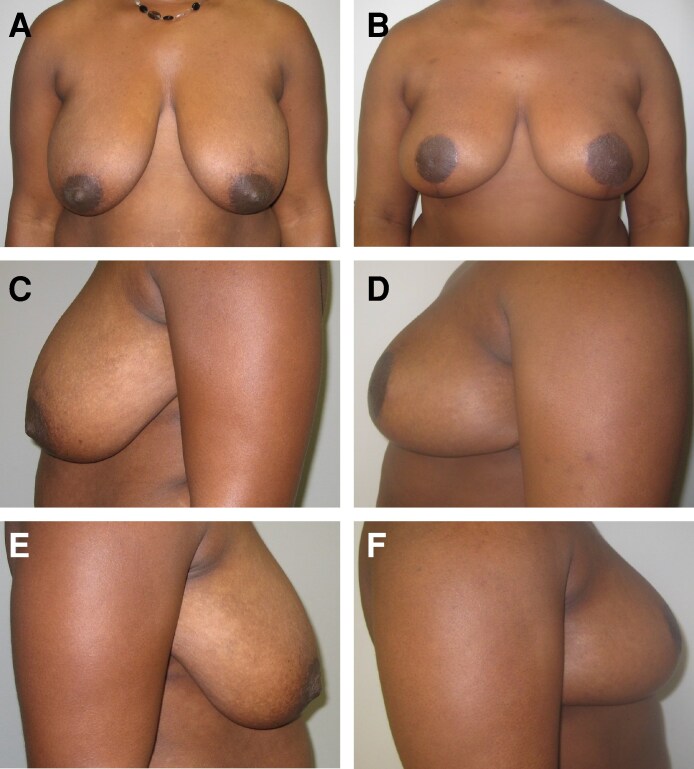
Case study 1. Representative preoperative photographs of the front (A), left-side (C), and right-side (E) views of a 42-year-old African American female who had lost weight with a GLP-1 RA prior to undergoing aesthetic breast reduction. Representative postoperative (1.5 years) photographs of the front (B), left-side (D), and right-side (F) views of the patient following aesthetic breast surgery.

A 42-year-old African American woman (Figure 2A–C) with a medical history of type 2 diabetes mellitus presented with grade 2 breast ptosis and mammary hypertrophy. The patient had lost weight (starting weight: 205 lb; stable weight: 175 lb) with a GLP-1 RA (Mounjaro [tirzepatide injection]; Eli Lilly and Company, Indianapolis, IN) and maintained a stable weight for 1 year prior to aesthetic breast surgery. The surgeon (M.N.) qualitatively determined that the patient had excellent skin quality and confirmed that the patient was an excellent candidate for aesthetic breast surgery. The patient stopped taking the GLP-1 RA 1 month prior to the aesthetic breast reduction procedure and resumed use 1 month following surgery. The patient had an 8-mm skin scab; no other adverse outcomes were observed, and the patient was satisfied with her procedure 1.5 years following the operation (Figure 2D–F).

### Case Study 2

**Figure 3.**
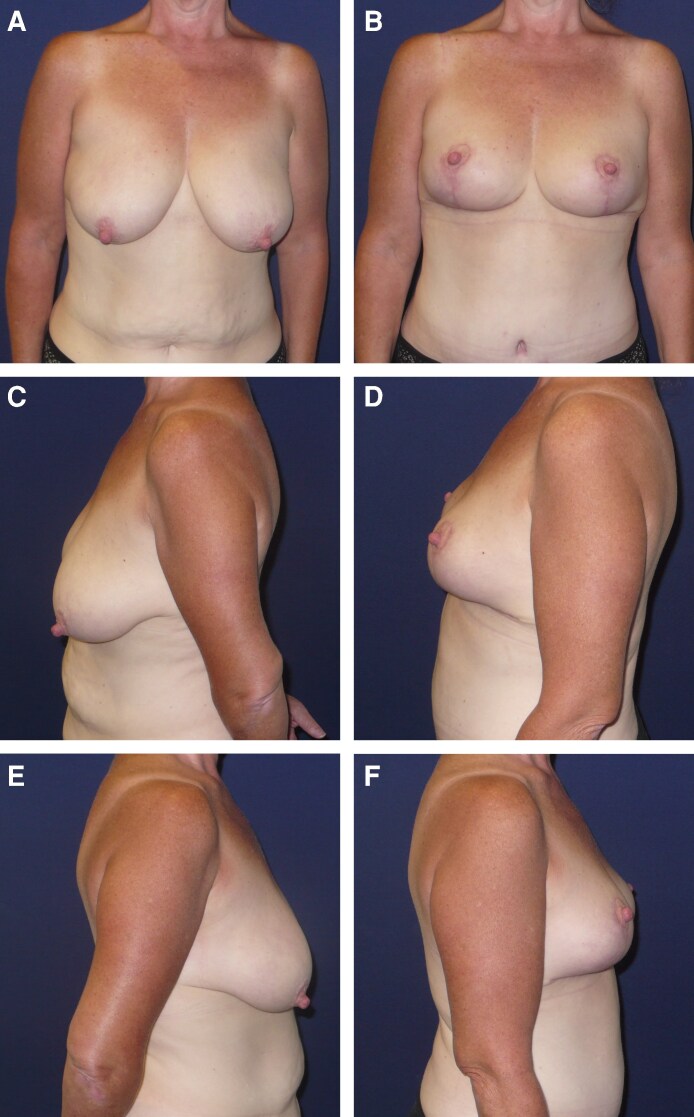
Case study 2. Representative preoperative photographs of the front (A), left-side (C), and right-side (E) views of a 50-year-old Caucasian woman who had lost weight with a GLP-1 RA prior to undergoing combined breast reduction/lift and abdominoplasty. Representative postoperative (6 months) photographs of the front (B), left-side (D), and right-side (F) views of the patient following aesthetic breast surgery.

A 50-year-old White woman (Figure 3A–C) with a medical history of metabolic syndrome, prediabetes, carpal tunnel release, and a prior hysterectomy presented with breast ptosis. The patient had lost weight (starting weight: ≈213 lb; stable weight: ≈136 lb) with a GLP-1 RA (Ozempic [semaglutide injection]; Novo Nordisk, Plainsboro, NJ) and maintained a stable weight for 1 year prior to aesthetic breast surgery. The patient stopped taking the GLP-1 RA 2 weeks prior to the procedure (combined breast reduction/lift and abdominoplasty) and resumed use 1 week following surgery. No adverse outcomes were observed, and the patient was satisfied with her procedure 6 months following the operation (Figure 3D–F).

## FUTURE AREAS OF RESEARCH

Further research is needed to evaluate aesthetic breast surgery in patients with weight loss resulting from the use of GLP-1 RAs. Future studies may investigate whether there are differences between patients who have lost weight with GLP-1 RAs, patients who have lost weight with other methods (eg, bariatric surgery or lifestyle changes), or patients who have not received any weight loss treatments. The results of these studies may impact aesthetic breast surgery considerations, such as how long a patient should maintain weight loss prior to aesthetic breast surgery, the type of procedure performed, and the optimal type, size, and position of breast implants that may be utilized. As we learn more about the long-term efficacy and limitations of GLP-1 RA treatment for weight loss, it is important that plastic surgeons keep up-to-date on specific effects on aesthetic surgery outcomes and are ready to modify their surgical choices and techniques to address these challenges to ensure that these patients achieve stable and predictable results from aesthetic breast surgery.

## CONCLUSIONS

As the use of GLP-1 RAs continues to increase, the number of patients who seek aesthetic procedures after weight loss is consequently expected to grow. In this narrative review, the authors recommend discussing the potential risks of aesthetic breast surgery following weight loss with patients during preoperative counseling, regardless of the weight loss mechanism used. Surgeons should be aware of which patients may be poor candidates for aesthetic surgery following massive weight loss, such as those with high BMI or poor overall medical condition, active smokers, those receiving anticoagulants, or those who have unrealistic expectations for aesthetic outcomes.
